# Transcatheter Edge‐to‐Edge Repair With MitraClip G4 System Compared With PASCAL System in Patients With Functional Mitral Regurgitation: A Propensity Score‐Matched Analysis

**DOI:** 10.1161/JAHA.125.044698

**Published:** 2026-08-27

**Authors:** Paula Ackermann, Suzanne de Waha, Wolfgang Otto, Johannes Rotta detto Loria, Antonia van Kampen, Sergey Leontyev, Jörg Ender, Sebastian Rosch, Mohamed Abdel‐Wahab, Holger Thiele, Philipp Lurz, Michael A. Borger, Tobias Kister, Thilo Noack

**Affiliations:** ^1^ Department of Cardiac Surgery Heart Center Leipzig at University of Leipzig Leipzig Germany; ^2^ Department of Rhythmology University Hospital Schleswig‐Holstein Lübeck Germany; ^3^ Department of Cardiology Heart Center Leipzig at University of Leipzig Leipzig Germany; ^4^ Department of Anesthesiology Heart Center Leipzig at University of Leipzig Leipzig Germany; ^5^ Department of Cardiology, Cardiology I University Medical Center of the Johannes Gutenberg‐University Mainz Mainz Germany

**Keywords:** functional mitral regurgitation, heart failure, mitral valve intervention, Transcatheter edge‐to‐edge repair, Valvular Heart Disease, Catheter-Based Coronary and Valvular Interventions

## Abstract

**Background:**

Functional mitral regurgitation (FMR), resulting from left ventricular dysfunction or left atrial dilation, is associated with heart failure and increased mortality. Mitral transcatheter edge‐to‐edge repair is an established treatment for patients with severe symptomatic FMR who are at high surgical risk. However, comparative data on the 2 most commonly used mitral transcatheter edge‐to‐edge repair systems, MitraClip G4 and PASCAL, remain limited. This study compared the safety and efficacy of both devices in patients with FMR.

**Methods:**

Consecutive patients with severe symptomatic FMR who underwent mitral transcatheter edge‐to‐edge repair using MitraClip G4 or PASCAL between 2021 and 2023 at a single center were included. Coprimary end points were 1‐year survival and freedom from heart failure hospitalization. Secondary end points included MR reduction and New York Heart Association functional class. Propensity score matching was performed to adjust for baseline differences.

**Results:**

The study included 319 patients, of whom 150 (47.0%) were treated with MitraClip G4 and 169 (53.0%) with PASCAL. After propensity score matching, 116 matched pairs were analyzed. Procedural success, defined as MR grade ≤2, was achieved in 98.1% of the MitraClip G4 group and 97.3% of the PASCAL group (*P*=0.61). At 1 year, no significant differences were observed between groups regarding residual MR (*P*=0.83), New York Heart Association class (*P*=0.41), or freedom from heart failure hospitalization (*P*=0.41). Estimated 1‐year survival was 86.7% for MitraClip G4 and 87.8% for PASCAL (*P*=0.84).

**Conclusions:**

In patients with FMR, MitraClip G4 and PASCAL demonstrated similarly high procedural success, durable MR reduction, and comparable clinical outcomes at 1 year.

**Nonstandard Abbreviations and Acronyms**
DMR: degenerative mitral regurgitation
FMR: functional mitral regurgitation
MR: mitral regurgitation
M‐TEER: mitral valve transcatheter‐edge‐to‐edge‐repair
MV: mitral valve

**Clinical Perspective**

**What Is New?**

- This study provides real‐world evidence of the 2 major contemporary mitral transcatheter edge‐to‐edge repair technologies, MitraClip G4 and PASCAL, specifically in patients with functional mitral regurgitation.

**What Are the Clinical Implications?**

- For patients with severe symptomatic functional mitral regurgitation at high surgical risk, clinicians can expect comparable effectiveness and safety with either MitraClip G4 or PASCAL.
- These results endorse a device‐neutral approach to mitral transcatheter edge‐to‐edge repair, enabling clinicians to choose the system best suited to individual anatomical features, leaflet morphology, and operator preference rather than outcome concerns.

Mitral regurgitation (MR) is a common heart valve disease characterized by retrograde flow from the left ventricle (LV) into the left atrium, leading to LV volume overload, heart failure, and eventually death. MR can result from abnormalities of the mitral valve (MV) or subvalvular apparatus, classified as primary or degenerative MR (DMR), or result from LV dysfunction or left atrium dilation, referred to as secondary or functional MR (FMR).^1^ FMR carries a particularly poor prognosis compared with DMR, with higher mortality rates and more challenging management due to the underlying cardiomyopathy and complex pathophysiology.^2^ Transcatheter edge‐to‐edge repair of the MV (M‐TEER) is an intervention that approximates the anterior and posterior MV leaflets using a clip device. It is a treatment option for patients with severe symptomatic MR and elevated surgical risk who remain refractory to optimal guideline‐directed medical therapy.^3^, ^4^ However, M‐TEER in FMR presents unique technical challenges, including restrictive leaflet motion, large coaptation gaps, and tethering forces that can complicate device deployment and optimal leaflet approximation.^5^ Further, studies have demonstrated a strong correlation between the degree of MR reduction achieved and clinical outcomes, with greater residual MR reduction associated with improved survival and symptom relief.^6^, ^7^ This relationship underscores the critical importance of optimal device selection and procedural technique. The safety and efficacy of the 2 leading systems, MitraClip (Abbott Laboratories, Chicago, IL, USA) and PASCAL (Edwards Lifesciences, Irvine, CA, USA), have been previously demonstrated.^8^, ^9^ Comparative evidence between the 2 devices in patients with FMR is currently limited to observational studies, small cohorts, short follow‐up durations, and heterogenous patients including both DMR and FMR.^10^, ^11^, ^12^, ^13^, ^14^, ^15^, ^16^, ^17^, ^18^ The aim of this study is therefore to provide a comprehensive comparative analysis of patients with FMR undergoing M‐TEER with either the MitraClip G4 or PASCAL device.

## METHODS

In this single center observational study, all consecutive patients with FMR undergoing M‐TEER using the MitraClip G4 system (Abbott Laboratories, Chicago, IL, USA) or the PASCAL system (Edwards Lifesciences, Irvine, CA, USA) between 2021 and 2023 at the Heart Center Leipzig at Leipzig University were evaluated. Implanted device types included the fourth‐generation MitraClip (NT, XT, NTW, XTW) and second‐generation PASCAL (P10, ACE). Inclusion criteria were age ≥18, symptomatic moderate to severe FMR despite optimal guideline‐directed medical therapy, and M‐TEER using either MitraClip G4 or PASCAL. Exclusion criteria were unfavorable MV anatomy for M‐TEER, prior MV interventions or surgery, and nonelective M‐TEER.

This study complied with the principles of the Declaration of Helsinki and was approved by the local ethics committee (No. 168/21‐ek) of the University of Leipzig, Faculty of Medicine. Written informed consent was obtained from all patients. The data that support the findings of this study are available from the corresponding author upon reasonable request.

### Preprocedural Workup and Procedure

The implantation procedures for the MitraClipG4 and PASCAL devices have previously been described.^19^, ^20^ Surgical or transcatheter treatment options were individually discussed by an interdisciplinary heart team. The choice between MitraClip G4 or PASCAL device was at the discretion of the operator based on established anatomically suitability criteria.^5^ Large mitral valve area and broad gap size rather opted for the use of M‐TEER devices with extended arms like XT, XTW and P10.^18^ In patients with occurrence of commissural jets and thin leaflet tissue smaller and more flexible devices like NT, NTW and PASCAL Ace should be implanted.^19^ All M‐TEER procedures were performed in hybrid operating rooms by an experienced team of interventional cardiologists, cardiac surgeons, and cardiac anesthesiologists.

### Definitions and Outcomes

All patients underwent transthoracic and transesophageal echocardiography at baseline. MR severity was assessed using regurgitant volume, vena contracta width, and effective regurgitant orifice area, as previously described.^21^ Ventricular FMR was characterized by LV dysfunction and mitral annular dilation in the presence of reduced LV systolic function or wall motion abnormalities as well as morphologically normal MV leaflets with restricted motion. Atrial FMR was defined by normal LV size, left atrium enlargement, and left annular dilation in the presence of preserved systolic LV function and normal MV leaflet morphology. The diagnosis was based on a comprehensive echocardiographic assessment, considering the possibility of mixed causes or late‐stage disease progression (eg, LV enlargement due to chronic severe atrial FMR).

The coprimary end points were 1‐year survival and freedom from hospitalization for heart failure. Secondary outcomes, reported according to the Mitral Valve Academic Research Consortium criteria,^12^ included procedural success and MR reduction as well as peri‐ and postprocedural events (stroke, major cardiovascular events, bleeding, acute kidney injury, valve‐related dysfunction and severe hypotension or acute heart failure). Follow‐up was performed at hospital discharge and 1 year. Examinations at 1‐year follow‐up included transthoracic echocardiography, New York Heart Association class grading, and assessment of hospitalization for heart failure.

### Statistical Analysis

Continuous variables were reported as mean ± SD if normally distributed or as median with interquartile range (IQR) if not normally distributed. Comparisons were performed using the unpaired *t*‐test for normally distributed data and Wilcoxon rank‐sum test for nonnormally distributed data. Categorical variables were reported as counts with percentages, analyzed using the chi‐square or Fisher's exact test. Kaplan–Meier survival curves were computed to analyze time‐to‐event outcomes. Hazard ratios (HR) with corresponding 95% CIs were calculated using Cox proportional hazards models.

Propensity score matching was performed using a nearest‐neighbor algorithm with a caliper width of 0.2 SDs of the logit of the propensity score and a 1:1 matching ratio to account for baseline differences between the 2 groups. A total of 9 clinically relevant baseline covariates were selected based on prior literature and clinical expertise. After matching, 116 well‐matched pairs were identified from the initial cohort of 150 and 169 patients, respectively. Balance assessment demonstrated standardized mean differences <0.10 for 4 variables and <0.15 for the remaining variables, indicating acceptable covariate balance between groups (Figure S1). Time‐to‐event analyses in the matched cohort were performed using Cox proportional hazards models stratified by matched pairs to account for the dependency within matched observations. As a sensitivity analysis, a multivariable Cox regression model including device type and the Society of Thoracic Surgeons score was performed in the unmatched cohort.

Calculations were performed using the R package MatchIt.^22^

## RESULTS

### Baseline Characteristics

Between 2021 and 2023, a total of 319 patients were included in our single‐center FMR registry, with 150 patients (47.0%) undergoing M‐TEER with the MitraClip G4 and 169 patients (53.0%) receiving a PASCAL device. Baseline characteristics are displayed in Table 1. Patients were at increased surgical risk (Society of Thoracic Surgeons score: MitraClip G4 4.9 [IQR, 3.3–7.8] versus PASCAL 4.5 [IQR, 2.7–8.0], *P*=0.52).

**Table 1:** Baseline Characteristics

	Unmatched			Matched		
	MitraClip G4	PASCAL	*P* value	MitraClip G4	PASCAL	*P* value
	No.=150	No.=169		No.=116	No.=116	
Baseline variables						
Age, y	77 (70–81)	77 (70–81)	0.81	77 (71–82)	77 (70–81)	0.86
Male sex, n (%)	86 (57.3)	87 (51.5)	0.35	61 (52.6)	61 (52.6)	1.00
Body mass index, kg/m^2^	26 (24–31)	26 (23–31)	0.71	26 (24–30)	26 (23–31)	0.95
Arterial hypertension, n (%)	133 (88.7)	149 (88.2)	1.00	105 (90.5)	102 (87.9)	0.67
Diabetes, n (%)	48 (32.0)	47 (27.8)	0.49	32 (27.6)	30 (25.9)	0.88
Hyperlipidemia, n (%)	103 (68.7)	125 (74.0)	0.36	78 (67.2)	84 (72.4)	0.48
Coronary artery disease, n (%)	89 (59.3)	93 (55.0)	0.51	68 (58.6)	60 (51.7)	0.36
Prior myocardial infarction, n (%)	36 (24.0)	43 (25.4)	0.87	26 (22.4)	33 (28.4)	0.36
Prior stroke/transient ischemic attack	13 (8.7)	12 (7.1)	0.76	9 (7.8)	8 (6.9)	1.00
Prior coronary artery bypass graft	22 (14.7)	29 (17.2)	0.65	14 (12.1)	20 (17.2)	0.35
Chronic obstructive pulmonary disease, n (%)	22 (14.7)	38 (22.5)	0.10	15 (12.9)	20 (17.2)	0.46
Pulmonary hypertension, n (%)	92 (61.3)	96 (56.8)	0.48	70 (60.3)	68 (58.6)	0.90
Chronic kidney disease, n (%)	92 (61.3)	113 (66.9)	0.36	66 (56.9)	75 (64.7)	0.28
Dialysis, n (%)	12 (8.0)	6 (3.6)	0.14	7 (6.0)	5 (4.3)	0.77
Peripheral arterial disease, n (%)	16 (10.7)	20 (11.8)	0.88	12 (10.3)	12 (10.3)	1.00
Operative risk prediction scores						
European System for Cardiac Operative Risk Evaluation	12.7 (8.0–21.0)	13.6 (8.4–24.4)	0.50	12.3 (7.4–19.4)	12.9 (8.0–22.0)	0.47
Society of Thoracic Surgeons score	4.8 (3.3–8.0)	4.8 (3.4–8.4)	0.80	4.9 (3.3–7.8)	4.5 (2.7–8.0)	0.52
New York Heart Association class, n (%)			0.10			0.73
I	0 (0)	0 (0)		0 (0)	0 (0)	
II	29 (20.6)	19 (11.7)		19 (16.4)	16 (13.8)	
III	86 (61.0)	112 (68.7)		77 (66.4)	76 (65.5)	
IV	26 (18.4)	32 (19.6)		20 (17.2)	24 (20.7)	

Preprocedural echocardiographic parameters are summarized in Table 2.

**Table 2:** Preprocedural Echocardiographic Parameters

	Unmatched			Matched		
	MitraClip G4	PASCAL	*P* value	MitraClip G4	PASCAL	*P* value
	No.=150	No.=169		No.=116	No.=116	
Left ventricular ejection fraction, %	46 (31–59)	42 (30–55)	0.24	43 (34–52)	45 (36–56)	1.00
MR cause, n (%)			0.38			0.58
Atrial FMR	77 (51.3)	75 (44.4)		61 (52.6)	54 (46.6)	
Ischemic V‐FMR	47 (31.3)	56 (33.1)		34 (29.3)	41 (35.3)	
Nonischemic V‐FMR	26 (17.3)	38 (22.5)		21 (18.1)	21 (18.1)	
Preinterventional MR grade, n (%)			0.26			0.19
None	0 (0)	0 (0)		0 (0)	0 (0)	
I	0 (0)	0 (0)		0 (0)	0 (0)	
II	18 (12.0)	29 (17.2)		13 (11.2)	21 (18.1)	
III	132 (88.0)	140 (82.8)		103 (88.8)	95 (81.9)	
Effective regurgitant orifice area, cm^2^	0.3 (0.2–0.4)	0.3 (0.2–0.4)	0.12	0.3 (0.2–0.4)	0.3 (0.2–0.4)	0.38
Mitral valve pressure gradient mean, mm Hg	2.0 (1.4–3.0)	2.0 (1.4–3.0)	0.98	2.0 (1.1–3.0)	2.0 (1.5–2.9)	0.64

MR indicates mitral regurgitation; and V‐FMR, ventricular functional mitral regurgitation.

### Procedural Characteristics

Procedural variables are outlined in Table 3. The MitraClip G4 group had a shorter fluoroscopic time compared with the PASCAL group (*P*=0.02), and total procedural times were comparable (*P*=0.20). No patient required emergency cardiac surgery or prolonged hemodynamic and respiratory support.

**Table 3:** Procedural Characteristics

	Unmatched			Matched		
	MitraClip G4	PASCAL	*P* value	MitraClip G4	PASCAL	*P* value
	No.=150	No.=169		No.=116	No.=116	
Procedural time, min	56 (38–75)	58 (45–82)	0.23	55 (40–70)	60 (45–85)	0.20
Fluoroscopic time, min	16 (11–23)	18 (12–25)	0.04	16 (11–22)	20 (12–30)	0.02
Implantation of ≥2 devices, n (%)	45 (30.0)	59 (34.9)	0.42	34 (29.3)	43 (37.1)	0.27
Life‐threatening bleeding, n (%)	0 (0)	1 (0.9)	1.00	0 (0)	0 (0)	‐
Vascular complications, n (%)	0 (0)	1 (0.9)	1.00	0 (0)	0 (0)	‐
Cardiac perforation, n (%)	0 (0)	0 (0)	‐	0 (0)	0 (0)	‐
Significant residual left‐to‐right shunt, n (%)	1 (1.5)	2 (1.7)	1.00	1 (1.9)	2 (2.3)	1.00
Valve‐related dysfunction, migration, thrombosis or other complication, n (%)	0 (0)	0 (0)	‐	0 (0)	0 (0)	‐
Single leaflet detachment, embolization or dehiscence, n (%)	0 (0)	0 (0)	‐	0 (0)	0 (0)	‐
Conversion to surgery, n (%)	0 (0)	0 (0)	‐	0 (0)	0 (0)	‐
Postinterventional mitral regurgitation grade, n (%)			0.31			0.06
None	12 (8.1)	13 (7.7)		12 (10.4)	4 (3.5)	
I	117 (78.5)	121 (72.0)		89 (77.4)	90 (78.3)	
II	19 (12.8)	29 (17.3)		14 (12.2)	18 (15.7)	
III	1 (0.7)	5 (3.0)		0 (0)	3 (2.6)	
Mitral valve pressure gradient mean, mm Hg	3.0 (2.0–3.0)	3.0 (2.0–3.5)	0.33	3.0 (2.0–3.0)	3.0 (2.0–4.0)	0.12

### Clinical Outcome at Hospital Discharge

Postprocedural outcomes are presented in Table 4. MR grade ≤2 was achieved in 98.1% of the MitraClip G4 group and 97.3% of the PASCAL group (*P*=0.61). The mean transvalvular gradient at discharge was similar between the groups (MitraClip G4: 3.0 mm Hg [IQR, 2.4–4.0] versus PASCAL: 3.0 mm Hg [IQR, 2.5–4.0], *P*=0.91). Before hospital discharge, 1 patient treated with MitraClip G4 died due to septic shock.

**Table 4:** Postprocedural Outcomes

	Unmatched			Matched		
	MitraClip G4	PASCAL	*P* value	MitraClip G4	PASCAL	*P* value
	No.=150	No.=169		No.=116	No.=116	
Echocardiographic outcome at discharge						
Mitral regurgitation grade, n (%)			0.22			0.61
None	3 (2.1)	8 (5.0)		3 (2.7)	3 (2.7)	
I	98 (67.6)	117 (72.7)		77 (69.4)	85 (75.9)	
II	39 (26.9)	30 (18.6)		29 (26.1)	21 (18.8)	
III	5 (3.5)	6 (3.7)		2 (1.8)	3 (2.7)	
Mitral valve pressure gradient mean at discharge, mm Hg	3.0 (2.4–4.0)	3.0 (2.1–4.0)	0.31	3.0 (2.4–4.0)	3.0 (2.5–4.0)	0.91
Clinical outcome						
Stroke, n (%)	0 (0)	0 (0)	‐	0 (0)	0 (0)	‐
Myocardial infarction, n (%)	1 (1.5)	0 (0)	0.37	1 (1.9)	0 (0.0)	0.39
In‐hospital mortality, n (%)	0 (0)	1 (3.1)	1.00	0 (0)	0 (0)	‐
Length of hospital stay, d	6 (3–13)	5 (3–12)	0.78	6 (3–11)	6 (3–13)	0.85

### Clinical Outcome at 1 Year

Clinical follow‐up at 1 year was completed in 93.1% of MitraClip G4 group and 94.8% of PASCAL group. One‐year echocardiographic results are displayed in Figure 1 and Table 5. There was no difference regarding MR ≤2 (97.7% versus 98.1%, *P*=0.83). Estimated 1‐year freedom from hospitalization for heart failure did not differ between groups (92.3% versus 95.3%, HR, 0.62 [95% CI, 0.20–1.96], *P*=0.41; Figure 2A). Estimated 1‐year survival was 86.7% in the MitraClip G4 group and 87.8% in the PASCAL group (HR, 0.93 [95% CI, 0.45–1.91], *P*=0.84; Figure 2B). Consistent with the primary analysis, device type was not associated with all‐cause mortality (PASCAL versus MitraClip G4: HR, 0.91, 95% CI, 0.54–1.51, *P*=0.71).

**Figure 1:**
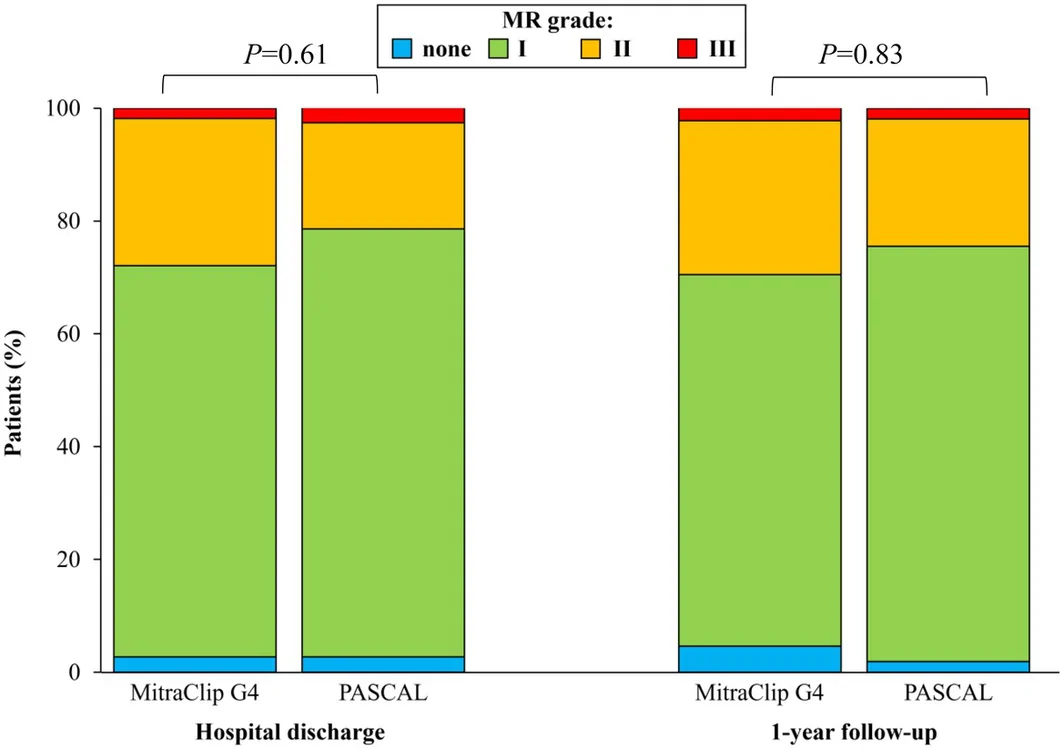
Mitral regurgitation grade at hospital discharge and 1‐year follow‐up in the matched cohort. MR indicates mitral regurgitation.

**Table 5:** Outcomes at 1 Year

	Unmatched			Matched		
	MitraClip G4	PASCAL	*P* value	MitraClip G4	PASCAL	*P* value
	No.=150	No.=169		No.=116	No.=116	
Echocardiographic outcome at 1 y						
Mitral regurgitation grade, n (%)			0.32			0.83
None	3 (5.9)	1 (1.3)		2 (4.6)	1 (1.9)	
I	32 (62.7)	56 (74.7)		29 (65.9)	39 (73.6)	
II	13 (25.5)	16 (21.3)		12 (27.3)	12 (22.6)	
III	3 (5.9)	2 (2.7)		1 (2.3)	1 (1.9)	
Mitral valve pressure gradient mean, mm Hg	3.3 (2.5–4.5)	3.0 (2.2–4.0)	0.36	3.2 (2.4–4.5)	3.0 (2.2–4.0)	0.53
Clinical outcome at 1 y						
New York Heart Association class, n (%)			0.41			0.41
I	3 (6.8)	4 (5.6)		2 (5.1)	2 (3.9)	
II	17 (38.6)	34 (47.2)		16 (41.0)	25 (48.1)	
III	24 (54.5)	31 (43.1)		21 (53.8)	22 (42.3)	
IV	0 (0)	3 (4.2)		0 (0)	3 (5.8)	

**Figure 2:**
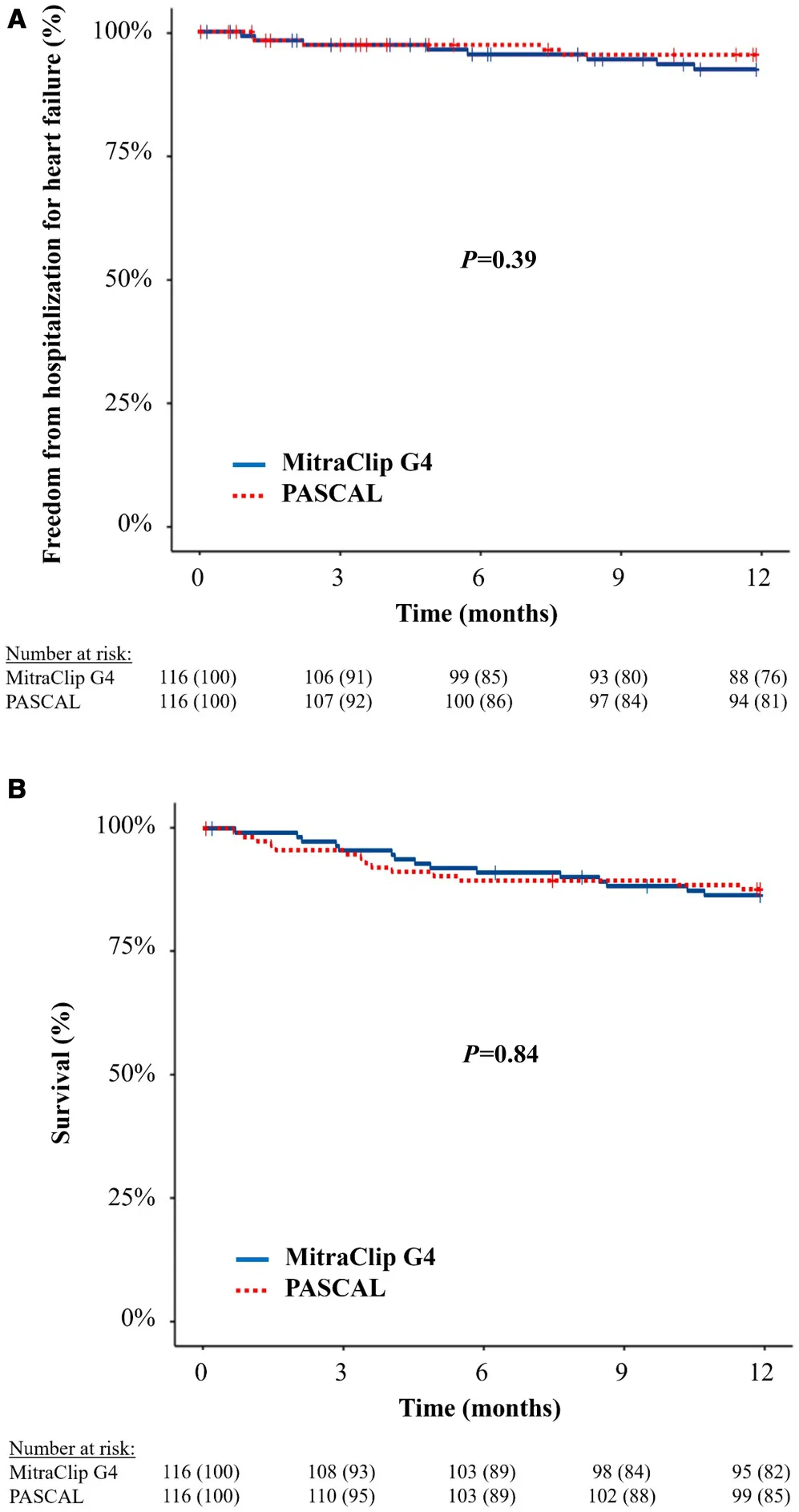
Time‐to‐first event analysis (from time of study entry) according to system used (MitraClip G4 versus PASCAL) after propensity‐score matching for (A) freedom from hospitalization for heart failure, and (B) all‐cause mortality.

## DISCUSSION

The current study presents outcomes of a single‐center cohort of patients with severe symptomatic FMR undergoing M‐TEER with either the MitraClip G4 or PASCAL system. The key findings can be summarized as follows: (1) both devices demonstrated high procedural success rates, significant MR reduction, and low rates of periprocedural complications; and (2) MR reduction was sustained over time, with comparable clinical outcomes at 1 year.

Although both M‐TEER systems share structural similarities, they exhibit some important differences, for example, the rigid cobalt‐chromium construction of the MitraClipG4 system in contrast to the woven nitinol scaffold of the PASCAL device. These distinctions may have procedural and clinical implications, making direct head‐to‐head comparisons between both M‐TEER systems of interest. The randomized controlled CLASP IID trial (Edwards PASCAL Transcatheter Valve Repair System Pivotal Clinical Trial) demonstrated noninferiority of the PASCAL system in terms of safety and efficacy compared with the MitraClip device.^10^, ^11^ However, this analysis was limited to patients with DMR, which is associated with more favorable short‐term and long‐term outcomes compared with patients with FMR. The ongoing randomized controlled CLASP IIF trial (Edwards PASCAL CLASP IID/IIF Pivotal Clinical Trial, NCT03706833) will expand the body of evidence specifically for patients with FMR, but patient enrollment determined by a central screening committee based on strict echocardiographic and clinical inclusion criteria may limit the generalizability of its finding to real‐world patients.

Currently, comparative data on MitraClip and PASCAL in patients with FMR remain limited to observational studies with small sample sizes, short follow‐up durations, and heterogenous cohorts including both patients with DMR and FMR.^10^, ^11^, ^12^, ^13^, ^14^, ^15^, ^16^, ^17^, ^18^ Our findings align with the most recent meta‐analysis of 7 studies encompassing both patients with DMR and FMR, which demonstrated comparable safety profiles and procedural success rates between the 2 devices, with no statistically significant differences in MR reduction.^23^ However, it has been hypothesized that the design of the PASCAL device, featuring greater flexibility due to the woven nitinol scaffold as well as broad convex paddles and a central spacer, may reduce mechanical leaflet traction, increase leaflet surface area, and mitigate restrictive diastolic opening. These characteristics could be particularly advantageous in the treatment of FMR.

Our group previously reported findings from an early experience of a smaller cohort of patients treated with either the MitraClip or PASCAL device between 2019 and 2020.^18^ This analysis suggested that PASCAL might provide more durable MR reduction and potentially preserve MV function more effectively. We expanded the previous analysis due to several limitations including a relatively small sample size (n=100), incompleteness of follow‐up, and the use of earlier‐generation M‐TEER devices. Consequently, these results may not be directly applicable to the latest‐generation MitraClip and PASCAL systems. Furthermore, due to the limited sample size, the clinical impact of these echocardiographic differences could not be fully determined. In the present analysis, all patients were treated with either the PASCAL P10/ACE system or the fourth‐generation MitraClip device, reflecting the latest advancements in M‐TEER technology. Moreover, increasing operator experience has been associated with higher rates of procedural success and lower rates of procedural complications.^24^ Notably, the overall procedure time (~60 minutes) was relatively short compared with previous studies, highlighting the expertise of the interventional team. Fluoroscopy time was significantly shorter in the G4 group, likely due to the operators' broader experience with MitraClip devices. The current analysis demonstrates that in the hands of these experienced operators, both devices achieve similar short‐term safety and efficiency with sustained MR reduction during the first year along with comparable clinical outcomes.

### Limitations

First, the current study is based on a single‐center analysis. The Heart Center Leipzig is one of the largest Heart Valve Centers in Europe and is equipped with all infrastructural resources required for optimal pre‐, peri‐, and postinterventional therapy. This may limit the extrapolation of the current results to other centers with lower M‐TEER volumes. Second, although this represents one of the largest cohorts to date comparing M‐TEER devices in patients with FMR, the overall sample size and number of events remain limited. This may reduce statistical power, particularly for detecting smaller differences between groups. Consequently, the findings should be interpreted with caution and require confirmation in larger, adequately powered studies. Third, the analysis is a nonrandomized retrospective study and is subject to the inherent limitations of observational data despite the use of propensity score matching. Furthermore, given the moderate sample size, propensity score matching reduces the effective sample size and may limit statistical power, while not fully eliminating residual confounding. This should be considered when interpreting the results.

## CONCLUSIONS

In summary, this study provides real‐world evidence supporting the use of both the MitraClip G4 and PASCAL devices in the treatment of severe symptomatic FMR. Both systems demonstrated high procedural success rates, durable MR reduction, and comparable clinical outcomes at 1 year, with no significant differences in mortality, heart failure hospitalization, or functional status. Although fluoroscopic times were slightly shorter with MitraClip G4, the clinical significance of this difference remains debatable. Future studies should focus on refining patient selection criteria, evaluating long‐term durability, and assessing the integration of M‐TEER within broader heart failure management strategies. As transcatheter MV therapies continue to evolve, a patient‐centered approach incorporating anatomical, clinical, and procedural considerations will be essential to optimizing outcomes. Ongoing randomized trials, extended follow‐up data, and real‐world registries will further enhance the understanding of M‐TEER therapy and its role in the contemporary management of MR.

## Sources of Funding

None.

## Disclosures

Suzanne de Waha is advisory board member for Zoll/Therox and has received speaker honoraria from Edwards Lifesciences. Dr Abdel‐Wahab discloses that his hospital receives speaker's honoraria and consultancy fees on his behalf from Medtronic, Boston Scientific, and Abbott. Dr Lurz has received institutional fees and research grants from Abbott Vascular, Edwards Lifesciences, and ReCor, honoraria from Edwards Lifesciences, Abbott Medical, Innoventric, ReCor, and Boehringer Ingelheim and has stock options with Innoventric. Dr Borger discloses that his hospital receives speakers' honoraria and consulting fees on his behalf from Edwards Lifesciences, Medtronic, Abbott, and CryoLife. Dr Thilo Noacks hospital receives speakers' honoraria on his behalf from Abbott, Edwards Lifesciences, and Medtronic. The remaining authors have no disclosures to report.

## Supporting information

Figure S1

STROBE Checklist
