# Supplementary material for: Transcatheter Edge‐to‐Edge Repair With MitraClip G4 System Compared With PASCAL System in Patients With Functional Mitral Regurgitation: A Propensity Score‐Matched Analysis
Source: J Am Heart Assoc. 2026 Aug 27;15(17):e044698. doi: 10.1161/JAHA.125.044698 (PMC13695825; doi:10.1161/JAHA.125.044698)
Supplement: Supplementary file 2 [file JAH3-15-e044698-s002.pdf]

Supplemental Material

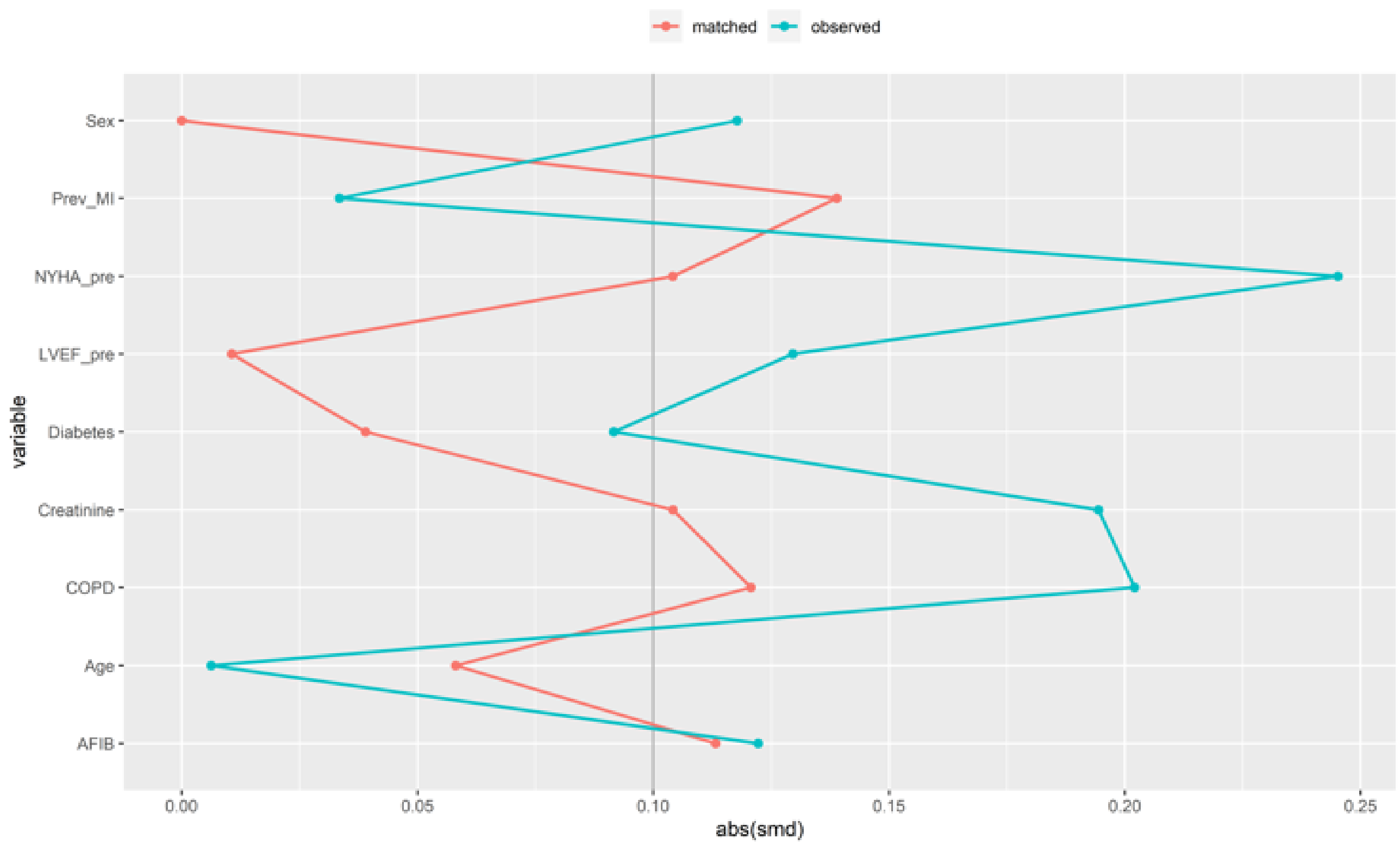

Figure S1
Love plots showing covariate balance before and after propensity score matching. Matched and observed cohort.
AFIB = atrial fibrillation; COPD = chronic obstructive pulmonary disease; LVEF_pre = left ventricular ejection fraction before intervention; PREV_MI = previous myocardial infarction; NYHA = New York Heart Association; SMD = standardized mean differences.
